# Reduced heart rate variability predicts fatigue severity in individuals with chronic fatigue syndrome/myalgic encephalomyelitis

**DOI:** 10.1186/s12967-019-02184-z

**Published:** 2020-01-06

**Authors:** Rosa María Escorihuela, Lluís Capdevila, Juan Ramos Castro, María Cleofé Zaragozà, Sara Maurel, José Alegre, Jesús Castro-Marrero

**Affiliations:** 1 Departament de Psiquiatria i Medicina Legal, Institut de Neurosciències, Universitat Autònoma de Barcelona, Facultat de Medicina, Avinguda Can Domènech, s/n, 08193 Bellaterra, Barcelona, Spain; 2 Laboratory of Sport Psychology, Department of Basic Psychology, Universitat Autònoma de Barcelona, Barcelona, Spain; 3 Department of Electronic Engineering, Biomedical and Electronic Instrumentation Group, Universitat Politécnica de Catalunya, Barcelona, Spain; 4 Clinical Research Department, Laboratorios Viñas, Barcelona, Spain; 5 Department of Medicine, Universitat Autònoma de Barcelona, Barcelona, Spain; 6 CFS/ME Unit, Vall d’Hebron University Hospital Research Institute, Universitat Autònoma de Barcelona, Passeig de Vall d’Hebron 119-129, 08035 Barcelona, Spain

**Keywords:** Autonomic dysfunction, Chronic fatigue syndrome, Fatigue, Heart rate variability, Myalgic encephalomyelitis

## Abstract

**Background:**

Heart rate variability (HRV) is an objective, non-invasive tool to assessing autonomic dysfunction in chronic fatigue syndrome/myalgic encephalomyelitis (CFS/ME). People with CFS/ME tend to have lower HRV; however, in the literature there are only a few previous studies (most of them inconclusive) on their association with illness-related complaints. To address this issue, we assessed the value of different diurnal HRV parameters as potential biomarker in CFS/ME and also investigated the relationship between these HRV indices and self-reported symptoms in individuals with CFS/ME.

**Methods:**

In this case–control study, 45 female patients who met the 1994 CDC/Fukuda definition for CFS/ME and 25 age- and gender-matched healthy controls underwent HRV recording-resting state tests. The intervals between consecutive heartbeats (RR) were continuously recorded over three 5-min periods. Time- and frequency-domain analyses were applied to estimate HRV variables. Demographic and clinical features, and self-reported symptom measures were also recorded.

**Results:**

CFS/ME patients showed significantly higher scores in all symptom questionnaires (p < 0.001), decreased RR intervals (p < 0.01), and decreased HRV time- and frequency-domain parameters (p < 0.005), except for the LF/HF ratio than in the healthy controls. Overall, the correlation analysis reached significant associations between the questionnaires scores and HRV time- and frequency-domain measurements (p < 0.05). Furthermore, separate linear regression analyses showed significant relationships between self-reported fatigue symptoms and mean RR (p = 0.005), RMSSD (p = 0.0268) and HFnu indices (p = 0.0067) in CFS/ME patients, but not in healthy controls.

**Conclusions:**

Our findings suggest that ANS dysfunction presenting as increased sympathetic hyperactivity may contribute to fatigue severity in individuals with ME/CFS. Further studies comparing short- and long-term HRV recording and self-reported outcome measures with previous studies in larger CFS/ME cohorts are urgently warranted.

## Background

Chronic fatigue syndrome (CFS), also known as myalgic encephalomyelitis (ME) is a debilitating and complex multisystem condition of unknown aetiology with high incapacitating potential due to severe and unexplained fatigue that cannot be alleviated by rest. Along with disabling fatigue, CFS/ME presents an array of core symptoms including autonomic dysfunction, unrefreshing sleep, cognitive impairment (sometimes called also “brain fog”), post-exertional malaise, and muscle pain (or myalgias) [1].

In medical research, heart rate variability (HRV) analysis is an objective and non-invasive tool that may play an important role in describing autonomic dysfunction in CFS/ME research and clinical practice, in tracking the natural fluctuations of autonomic function across various time spans, and in predicting the patients’ prognosis. Assessment of autonomic dysfunction by HRV analysis is easy to perform and offers the important advantage that it can track dynamic changes of cardiac autonomic function within minutes. Recently developed time- and frequency-domain analyses further enhance the ability of HRV analysis to track active changes in cardiovascular autonomic function. These analyses are likely to become part of future diagnostic criteria for CFS/ME and may serve as a surrogate end-point marker in clinical trials [2, 3].

Previous studies have provided strong evidence for autonomic nervous system (ANS) involvement in individuals with CFS/ME, including perturbations in the structure and function of brain (reflected by fMRI testing) [4–11]. In brief, cerebral blood flow volume changes and reduced HRV may lead to greater exacerbation of ANS symptoms in individuals with CFS/ME. In the current study, we performed both time and frequency domain HRV analyses to examine closely the relationship between the physiological parameters of HRV and patient-reported symptom measurements. Time-domain variables are statistical operations on R–R intervals that measure the dispersion of the individual cardiac cycle length around their mean. Frequency-domain parameters provide information on the different constituents (frequencies) of the heart rate signal and their relative intensity (power) [12]. There are some discrepancies in the literature about the behaviour of the HRV variables in CFS/ME individuals: Yamamoto et al. [13], for example, reported lower time- and frequency-domain parameters during head-up tilt in CFS/ME patients compared with healthy controls, but no differences in the baseline supine position. Yataco et al. [14] did not find differences either in the baseline supine state or in response to upright tilt. De Becker et al. [15] found differences in frequency domain parameters after tilting, but not in the supine position; and Frith et al. [16] found changes only in frequency domain variables measured at rest.

In this study we aimed: (1) to explore different diurnal HRV time and frequency domain parameters as potential biomarker in CFS/ME, and (2) to assess whether these HRV measures are associated with early self-reported symptoms in individuals with CFS/ME.

## Methods

### Participants’ characteristics

A prospective, cross-sectional, case–control cohort study of 45 consecutive females who met the 1994 CDC/Fukuda definition for CFS/ME [17] were recruited from a single outpatient tertiary referral centre (CFS/ME Unit, Vall d’Hebron University Hospital, Barcelona, Spain) from January 2014 through March 2015. Twenty-five matched non-fatigued healthy controls were also included through word-of-mouth and advertisements from the local community. All participants were of Caucasian descent, from the same geographical area, and had a sedentary life style at the time of study. Exclusion criteria for the study were previous or current diagnosis of autoimmune illnesses, multiple sclerosis, psychosis, major depression, cardiovascular disorders, haematological disorders, infectious diseases, sleep apnea or thyroid-related illnesses, pregnancy or breast-feeding, smoking, hormone-related drugs, or any symptoms that might be confused with those of CFS/ME.

### Setting and data collection

All eligible participants attended a first face-to-face interview in which demographic and clinical characteristics were collected along with patient-reported outcome measures. They were asked about their fatigue severity using the Fatigue Impact Scale (FIS-40), their autonomic dysfunction with both the abbreviated Composite Autonomic Symptom Score (COMPASS-31) and Neurovegetative Complaints Questionnaire (NCQ), their sleep problems using the Pittsburgh Sleep Quality Index (PSQI), and anxiety and depression through the Hospital Anxiety and Depression Scale (HADS). After the first interview, participants were set dates for the following additional HRV recording sessions. All records were done at the same location during working days.

### Measures

Participants were asked to fill out validated self-reported outcome measures as symptom assessment tools. The following measures were used to evaluate all participants under the supervision of two trained investigators (JC-M and JA) who oversaw participant compliance.

### Neurovegetative Complaints Questionnaire

The Neurovegetative Complaints Questionnaire (NCQ) was originally developed to measure neurovegetative, somatic and emotional complaints in post-concussive patients. The original questionnaire consisted of 28 items concerning headaches, problems with falling asleep, restlessness, chest pain, indigestion, slowness of working, sensitivity to light, effort, flushing, concentration, dyspnoea, preference for being left alone, tiredness, fainting, heart palpitations, noise, difficulty with doing two tasks simultaneously, preference for working at one’s own pace, dizziness, depression, wet hands, crying spells, altered libido, irritability, lack of initiative, awakening at night, defeatism, and not being appreciated by others. Participants had to indicate the frequency of occurrence of these symptoms on a 4-point Likert scale (1 = no, never; 2 = yes, sometimes; 3 = yes, regularly, and 4 = yes, often). Higher scores indicated more autonomic complaints [18].

### Fatigue Index Scale

The Fatigue Impact Scale (FIS-40) is a 40-item questionnaire designed to assess fatigue symptoms as part of an underlying chronic condition. It includes three domains reflecting the perceived feeling of fatigue: physical (10 items), cognitive (10 items) and psychosocial functions (20 items). Each item is scored from 0 (no fatigue) to 4 (severe fatigue). The overall score is calculated by adding together the responses to the 40 questions (ranging from 0 to 160). Higher scores indicate more functional limitations due to fatigue [19].

### Composite Autonomic Symptom Score

For measuring autonomic dysfunction, all participants were screened the Composite Autonomic Symptom Score (COMPASS-31), a 31-item questionnaire designed to evaluate the frequency and severity of autonomic function symptoms, grouped in six domains: orthostatic intolerance (4 items), vasomotor (3 items), secretomotor (4 items), gastrointestinal (12 items), bladder (3 items) and pupillomotor systems (5 items). Added together, the six domain scores provide a total COMPASS-31 score ranging from 0 to 100. Higher scores indicate more severe autonomic complaints [20].

### Pittsburgh Sleep Quality Index

The Pittsburgh Sleep Quality Index (PSQI) is a 19-item self-administrated questionnaire commonly used to assess sleep disturbances over a 1-month interval. Scores are acquired on each of seven domains of sleep quality: subjective sleep quality, sleep latency, sleep duration, habitual sleep efficiency, sleep disturbances, use of sleeping medication, and daytime dysfunction. Each domain is scored from 0 to 3 (0 = no problems and 3 = severe problems). The overall PSQI score ranges from 0 to 21 points, with scores of ≥ 5 indicating poorer sleep quality [21].

### Hospital Anxiety and Depression Scale

To assess anxiety and depression symptoms the Hospital Anxiety and Depression Scale (HADS) was used; a validated 14 item self-reported measure (seven items associated with anxiety symptoms and seven with depression) over the last week. Each item is scored on a 4-point Likert scale (e.g., 0 = as much as I always do; 1 = not quite so much; 2 = definitely not so much; and 3 = not at all) giving maximum subscale scores of 21 for depression and anxiety, respectively. Scores of 0–7 are interpreted as normal; scores of 8–10 reflect mild symptoms, 11–14 moderate, and 15–21 severe for either anxiety or depression. The global HADS score ranges from 0 (no anxiety/depression) to 42 (severe anxiety/depression symptoms) [22].

### Heart rate variability analysis

Heart rate variability was assessed between 3:00 pm and 6:00 pm in a semi-dark room maintained at a temperature between 20 and 24 °C during weekdays. At the start of each test session, all subjects were asked to abstain from alcoholic and caffeinated beverages, cigarette smoking, cardioactive medications and heavy exercise (physical/mental activity) for at least 48 h prior to testing, as their actions might alter their autonomic function. After 5 min of rest lying down, participants were asked to remain in supine position and still without speaking or making any movements, and HRV data were recorded continuously for 5 min of natural breathing. Participant’s session ratings of continuous heart rate (RR or beat-to-beat cardiac intervals) were collected and analysed with the FitLab^®^ system (HealthSportLab.com, Barcelona, Spain); an application that runs on a mobile device (iOS, Apple) connected via Bluetooth (BTv4) with a cardiac chest band (Polar Electro Oy, Kempele, Finland) and connected via wireless to a remote server. The system allows performance of individual HRV recordings in each session and checks data quality in real time. All participants performed the three 5-min HRV tests at rest in the supine position. The intervals between consecutive heartbeats (R–R) were continuously monitored over three 5-min periods on different days.

Heart rate variability analysis was performed for RR intervals in 5 min periods using the FitLab^®^ software, following the recommendations of the Task Force of the European Society of Cardiology and the North American Society of Pacing and Electrophysiology [23].

For the time domain analysis, the mean of RR intervals (mean RR), the standard deviation of all RR intervals (SDRR), the root mean square of differences (RMSSD) of successive RR intervals and the mean number of times an hour in which the change in successive normal sinus (RR) intervals exceeds 50 mseg (pNN50) were calculated. For frequency domain analysis, all RR series were resampled at 3 Hz using a cubic spline prior to the HRV analysis. Power–frequency analysis of the 5-min recordings was performed sequentially with a fast Fourier transform based on a non-parametric algorithm with a Welsh window after the ectopic-free data were detrended and resampled with a cubic spline interpolation [24]. The power in the very low frequency (VLF) band (0.0033–0.04 Hz), the low frequency (LF) band (0.04–0.15 Hz) and the high frequency (HF) band (0.15–0.40 Hz) were calculated from each 5-min spectrum by integrating the spectral power density in the respective frequency bands. Additional calculations included the LF/HF ratio, as well as the normalized LF and HF values (LFnu and HFnu, respectively). For the final analysis of each variable, the values obtained in the three independent recordings were averaged.

### Statistical analysis

Data were analysed using the IBM SPSS Statistics Package for Mac OS (version 23.0, SPSS Inc., Chicago, IL). Descriptive statistics and outcome measures for each item were shown as mean ± standard error of the mean. Calculation of HRV parameters was carried out with FitLab^®^ software (HealthSportLab.com, Barcelona, Spain) and MATLAB environment. Differences between unrelated groups were assessed with the Student’s t-test for independent samples, and non-parametric Mann–Whitney U test when appropriate. Pearson correlation analyses were used to test bivariate associations between questionnaires scores and HRV indices or between questionnaires scores themselves. Finally, to further evaluate the relative relationship between the most significant HRV parameters and patient-reported fatigue severity, we performed separate regression analyses in CFS/ME patients and healthy controls. The significance threshold was set at p < 0.05.

## Results

### Demographic and clinical characteristics of participants

Table 1 presents the demographic, clinical characteristics and baseline self-reported measures among the participants. There were no significant differences in age, BMI or SBP between ill and healthy controls. Heart rate and diastolic blood pressure were higher among ill than healthy controls (p = 0.002 and p = 0.022, respectively).

**Table 1:** Demographic and clinical characteristics of participants at time of recruitment

Variable	Healthy controls (n = 25)	CFS/ME (n = 45)	*p*-value^a^
Age (years)	44.96 ± 1.30	46.41 ± 0.84	N.S.
BMI (kg/m^2^)	23.77 ± 0.61	24.59 ± 0.69	N.S.
SBP (mmHg)	115.2 ± 2.15	121.2 ± 1.99	0.057
DBP (mmHg)	74.45 ± 1.56	79.56 ± 1.38	0.022
HR (bpm)	67.71 ± 1.93	74.72 ± 1.21	0.002
NCQ	0.40 ± 0.15	10.11 ± 0.28	< 0.001
FIS-40			
Global score (0–160)	17.12 ± 3.25	140.9 ± 1.79	< 0.001
Physical	4.60 ± 0.94	36.95 ± 0.39	< 0.001
Cognitive	4.48 ± 1.03	35.73 ± 0.66	< 0.001
Psychosocial	8.04 ± 1.46	68.27 ± 1.04	< 0.001
COMPASS-31			
Global score (0–100)	27.31 ± 2.42	80.10 ± 2.91	< 0.001
Orthostatic intolerance	2.52 ± 0.25	7.45 ± 0.31	< 0.001
Vasomotor	0.48 ± 0.21	1.93 ± 0.24	< 0.001
Secretomotor	0.76 ± 0.18	4.73 ± 0.18	< 0.001
Gastrointestinal	5.60 ± 0.74	13.45 ± 0.68	< 0.001
Bladder	0.32 ± 0.11	3.48 ± 0.32	< 0.001
Pupillomotor	2.96 ± 0.46	10.32 ± 0.55	< 0.001
PSQI			
Global score (0–21)	4.52 ± 0.63	15.05 ± 0.57	< 0.001
Subjective sleep quality	0.56 ± 0.12	2.23 ± 0.14	< 0.001
Sleep latency	0.72 ± 0.17	1.89 ± 0.16	< 0.001
Sleep duration	0.92 ± 0.17	2.05 ± 0.13	< 0.001
Habitual sleep efficiency	0.56 ± 0.22	1.95 ± 0.17	< 0.001
Sleep disturbances	1.04 ± 0.07	2.27 ± 0.13	< 0.001
Sleeping medication	0.28 ± 0.15	2.11 ± 0.19	< 0.001
Daytime dysfunction	0.44 ± 0.12	2.55 ± 0.11	< 0.001
HADS			
Global score (0–42)	5.15 ± 0.70	26.68 ± 1.41	< 0.001
Anxiety	3.96 ± 0.41	13.73 ± 0.73	< 0.001
Depression	1.16 ± 0.29	12.95 ± 0.68	< 0.001

Baseline self-reported measures scores (global and subscales) of symptoms, as explained in “Methods” section. Data are shown as mean ± SEM for each item

^a^Data were analyzed using Student’s t test for independent samples, and the Mann–Whitney U non-parametric test when appropriate

### Self-reported measures

The NCQ scores and the physical, cognitive, psychosocial domains and global FIS-40 scores were an average of eight times higher than those reported by the healthy controls (p < 0.001; Table 1). The higher scores on these measures were consistent with the CFS/ME diagnosis. Regarding the autonomic symptoms self-reported in the COMPASS-31 questionnaire, the CFS/ME patients reported increases in orthostatic intolerance, vasomotor, secretomotor, gastrointestinal, bladder, pupillomotor dysfunction (all p < 0.001) and overall COMPASS-31 score (p < 0.001) compared with the control subjects (Table 1). These results are also consistent with an increased number of autonomic symptoms reported in the NCQ (Table 1).

Regarding the patient-reported sleep symptoms in the PSQI questionnaire, the CFS/ME patients had higher scores for sleep quality, sleep latency, sleep duration, and sleep efficiency than controls (all p < 0.001; Table 1). CFS/ME patients also reported increased sleep disturbances, greater use of sleep medication and increased daytime dysfunction (all p < 0.001; Table 1), and their total PSQI score was three times higher than in controls (p < 0.001, Table 1). CFS/ME patients reported higher scores of anxiety and depression on the HADS questionnaire (a mean of 10–11 points more) than controls (p < 0.001, Table 1).

### Heart rate variability indices

**Fig. 1:**
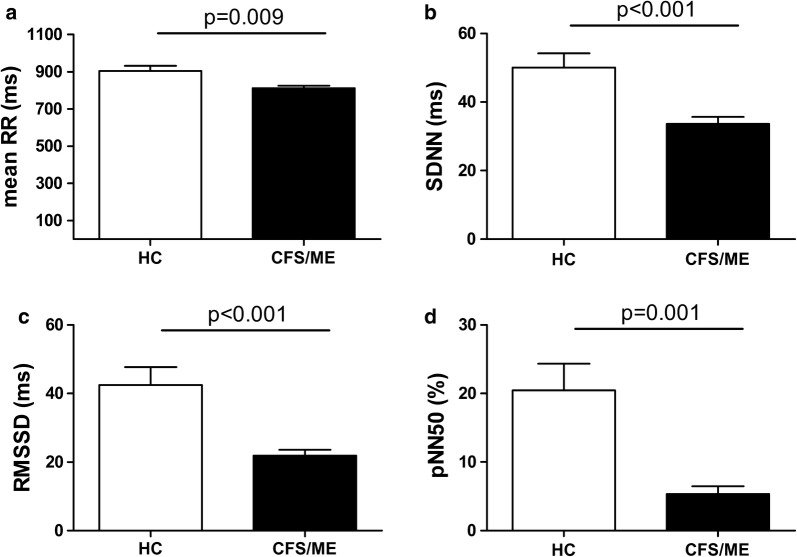
Comparison of the HRV time-domain parameters among the participants. Values are shown as mean ± SEM of **a** mean of RR intervals (mean RR), **b** standard deviation of all R–R intervals (SDNN), **c** root mean square of successive RR intervals differences (RMSSD), and **d** the mean number of times in an hour in which the change in successive normal sinus R–R intervals exceeds 50 ms (pNN50). Significance level is indicated above the horizontal line in each chart

**Fig. 2:**
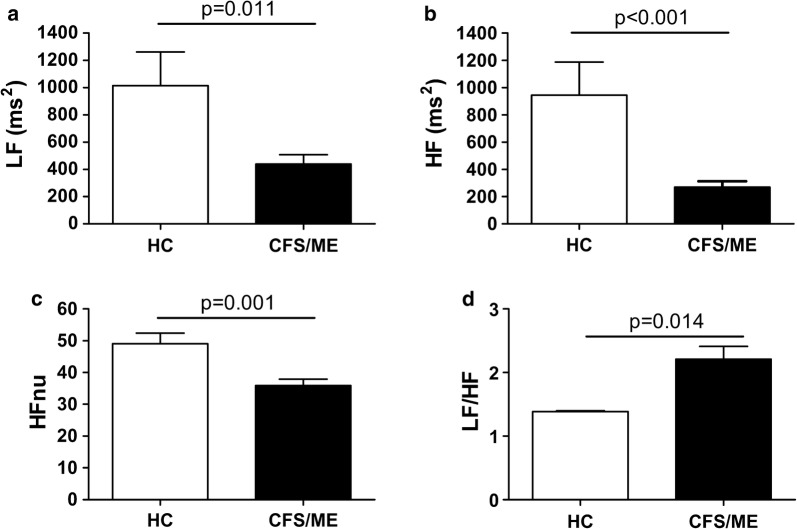
Comparison of the HRV frequency-domain measures in the study participants. Values are shown as mean ± SEM of **a** absolute power of the low frequency band (LF), **b** absolute power of the high frequency band (HF), **c** normalized HF value (HFnu) and **d** ratio of LF-to-HF power (LF/HF). Significance level is indicated above the horizontal line in each chart

Heart rate variability analysis showed significantly decreased values in CFS/ME patients compared with healthy controls for the time domain parameters: mean RR (Fig. 1a, p = 0.009), SDNN (Fig. 1b, p < 0.001), RMSSD (Fig. 1c, p < 0.001) and pNN50 (Fig. 1d, p < 0.001). HRV analysis for the frequency domain revealed significantly lower values for the parameters (LF, Fig. 2a, p = 0.011; HF, Fig. 2b, p < 0.001; HFnu Fig. 2c, p = 0.001), and higher LF/HF index (Fig. 2d, p = 0.014) in CFS/ME patients than in healthy controls.

### Correlation between clinical measures and HRV parameters

Table 2 displays the correlation analysis between self-reported symptoms and HRV domain indices, showing a negative association between the global FIS-40 score and the subscales with all HRV domain indices (p < 0.01), except for the LF/HF ratio with which the association was positive (p < 0.05). Regarding COMPASS-31, the domains for orthostatic intolerance, secretomotor and total score correlated negatively with all HRV indices (p < 0.01), except with LF/HF with which the correlation was positive (p < 0.05). The gastrointestinal domain correlated negatively with all HRV indices (p < 0.01), except for the LF/HF ratio which correlated positively (p < 0.05). The pupillomotor domain showed a negative correlation with all indices except for LF/HF index. The vasomotor domain presented a significant negative correlation with mean RR (p < 0.05); and the bladder item correlated negatively with HFnu (p < 0.05).

**Table 2:** Pearson correlation coefficients (r) between HRV parameters (both time- and frequency-domains) and self-reported measures in the sample

	Mean RR	SDRR	RMSSD	pNN50	LF	HF	LF/HF	HFnu
PSQI								
Sleep quality	− .244*	− .302*	− .302*	− .279*	− .226	− .275*	.174	− .203
Sleep latency	− .047	− .190	− .190	− .161	− .124	− .170	.188	− .152
Sleep duration	− .223	− .290*	− .295*	− .251*	− .180	− .320**	.213	− .216
Habitual sleep efficiency	− .182	− .288*	− .306*	− .285*	− .251*	− .308*	.284*	− .249*
Sleep disturbances	− .301*	− 286*	− .323**	− .291*	− .226	− .275*	.303*	− .283*
Sleeping medication	− .261*	− .407**	− .385**	− .380**	− .285*	− .322**	.130	− .190
Daytime dysfunction	− .297*	− .394**	− .394**	− .385**	− .277*	− .332**	.367**	− .403**
Global score	− .285*	− .405**	− .409**	− .381**	− .295*	− .373**	.302*	− .312**
NCQ	− .332**	− .378**	− .409**	− .399**	− .250*	− .358**	.341**	− .409**
FIS-40								
Physical	− .438**	− .442**	− .494**	− .498**	–.326**	− .435**	.309*	− .423**
Cognitive	− .440**	− .464**	− .507**	− .512**	–.322**	− .451**	.360**	− .476**
Psychosocial	− .439**	− .462**	− .501**	− .502**	–.337	− .438**	.324**	− .431**
Global score	− .442**	− .460**	− .504**	− .507**	–.332	− .443**	.332**	− .443**
HADS								
Anxiety	− .334**	− .347**	− .379**	− .377**	− .273*	− .339**	.137	− .283*
Depression	− .371**	− .407**	− .446**	− .435**	− .249*	− .377**	.308*	− .449**
Global score	− .367**	− .390**	− .429**	− .422**	− .271*	− .372**	.236	− .384**
COMPASS-31								
Orthostatic intolerance	− .367**	− .383**	− .390**	− .362**	− .283*	− .323**	.276*	− .314**
Vasomotor	− .293*	− .155	− .170	− .194	− .095	− .092	.060	− .186
Secretomotor	− .330**	− .379**	− .395**	− .396**	− .291*	− .319**	.271*	− .333**
Gastrointestinal	− .315**	− .278*	− .342**	− .360**	− .203	− .251*	.301*	− .356**
Bladder	− .183	− .181	− .239	− .229	− .133	− .225	.219	− .301*
Pupillomotor	− .322**	− .319**	− .347**	− .353**	− .189	− 294*	.192	− .289*
Global score	− .368**	− .359**	− .400**	− .397**	− .261*	− .317**	.307*	− .370**

Significance: *p < 0.05; **p < 0.01

The HADS-depression domain showed a negative correlation with all HRV indices (p < 0.01), and a positive correlation with LF/HF ratio (p < 0.05). Finally, HADS-anxiety domain showed a negative correlation (p < 0.05), but no correlation with LF/HF index.

On the PSQI questionnaire, sleep quality showed a negative correlation with the four indices of the HRV time domain analysis (p < 0.05), but only with the HF frequency domain parameter (p < 0.05). Sleep latency did not correlate with any HRV parameter. Sleep duration showed a negative correlation with the HRV time domain parameters (SDNN, RMSSD and pNN50; all p < 0.05), but only a negative correlation with HF for the frequency domain (p < 0.01). Habitual sleep efficiency correlated significantly negatively with all indices (p < 0.05), except with mean RR (no significant correlation), and showed a positive correlation with LF/HF index (p < 0.05). Sleep disturbances correlated negatively with all indices (p < 0.05) except for LF (no significant correlation), and positively with LF/HF (p < 0.05). Sleep medication correlated negatively with all indices (p < 0.05) except for LF/FH and HFnu (no significant correlation). Daytime dysfunction and overall PSQI score correlated negatively with all HRV parameters (p < 0.01); except for a significant positive correlation with LF/HF index (p < 0.01).

As showed in Table 2, all correlations were negative for the outcomes measures scores, except for LF/HF ratio, thus indicating that lower HRV domain values were associated with higher self-reported measure scores. However, a higher LF/HF index was associated with higher clinical symptom scores.

Interestingly, a closer look shows that FIS-40, HADS-depression, overall PSQI and NCQ questionnaires had indices lower than − 0.40 and significances greater than 0.01. In more in detail, the FIS-40 scores had correlation indices below − 0.423 with all HRV time-domain parameters (mean RR, SDNN, RMSSD and pNN50), and with the HF and HFnu frequency-domain parameters, thus suggesting a robust and consistent association between fatigue symptoms and these HRV domain parameters. HADS-depression had a correlation index below − 0.407 with SDNN, RMSSD and pNN50 time-domain parameters, and with HFnu index of the frequency domain. Finally, overall PSQI had a negative correlation with SDNN and RMSSD time domain (r = − 0.407 and r = − 0.409, respectively) but did not correlate with any frequency domain parameters.

**Fig. 3:**
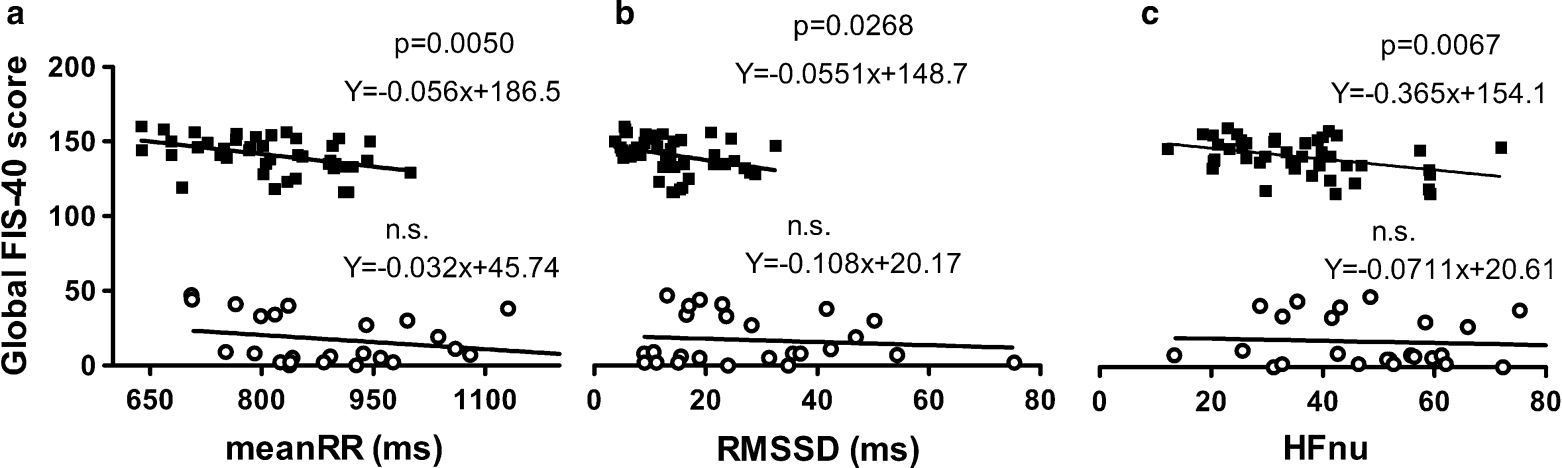
Correlation analysis between fatigue severity perception and HRV indices among the participants. Total FIS-40 score significantly correlated with **a** the mean RR, **b** the RMSSD, and **c** the HFnu among CFS/ME patients (black squares, upper regression lines) but not for those healthy controls (white circles, bottom regression lines). Significant correlations were found between HRV indices and presence of perceived fatigue among CFS/ME individuals (p < 0.01 for mean RR and HFnu, and p < 0.05 for RMSSD), but not in healthy controls

Finally, the simple linear regression analyses revealed clear differences among the study participants, since CFS/ME patients showed significant relationship between fatigue severity as measured by the FIS-40 and HRV domain parameters such as mean RR (Fig. 3a, r = − 0.056, p = 0.005), RMSSD (Fig. 3b, r = − 0.055, p = 0.027) and HFnu (Fig. 3c, r = − 0.365, p = 0.007). No such associations were found for each HRV domain in healthy controls.

Table 3 shows Pearson’s correlations between global scorings of self-reported questionnaires as PSQI, NCQ, COMPASS-31, and HADS. All correlations were positive, highly significant and higher than 0.71 (p < 0.01). Most correlation coefficients (r-values) were higher than 0.8. The r-value between FIS-40 and NCQ was 0.942 (Table 3).

**Table 3:** Pearson correlations between measures assessed in the sample

Measures	NCQ	FIS-40	HADS	COMPASS-31
PSQI	.824**	.813**	.729**	.816**
NCQ		.942**	.796**	.852**
FIS-40			.843**	.839**
HADS				.796**

Significance: **p < 0.001

## Discussion

This study, designed to identify HRV analysis-derived putative indices associated with self-reported measures in CFS/ME, provides the first evidence of a significant relationship between HRV and fatigue severity in this condition. The findings of this study showed a high association between the questionnaires scores themselves, indicating a close relationship among all the symptoms. A second finding was that all HRV indices (from frequency and temporal domain analyses), except for the LF/HF ratio, were negatively correlated with the self-reported questionnaire scores in both ill and control groups. This indicates that, as expected, low HRV is associated with high scores for fatigue, autonomic dysfunction, sleep quality, anxiety and depression symptoms. Finally, this study reveals a relationship of two HRV indices (RMSSD and HFnu) with fatigue symptoms; that is, low values of RMSSD (obtained from time domain HRV analysis) and low values of HFnu (obtained from frequency domain analysis) are specifically associated with high fatigue symptoms (as assessed by overall FIS-40 score) in the CFS/ME patients, but not in healthy controls. As this relationship did not appear between other HRV variables and other self-reported measures scores, we believe that this is the first evidence of an association between HRV changes and outcome measures in CFS/ME.

This study shows that HRV analysis is a clinically useful non-invasive tool for predicting fatigue severity in CFS/ME. Both the time- and frequency-domain indices were closely related to self-reported autonomic dysfunction, sleep quality, and anxiety and depression in clinical outpatients. CFS/ME patients had lower mean RR, SDNN, RMSSD and pNN50 than healthy controls, and lower LF, HF and HFnu but higher LF/HF. Even though the first set of these HRV parameters were obtained from the time domain analysis and the second set from the frequency domain analysis, they all indicate that CFS/ME patients showed decreased HRV associated with autonomic dysfunction, sleep quality and anxiety/depression symptoms. This is an important finding because the two types of variables point in the same direction, thus adding robustness to the results.

This is the first study to show this consistency between the different HRV indices. To some extent, our results corroborate those of previous studies. Yamamoto et al. [13] reported lower mean RR, but not SDNN, in CFS/ME patients than in matched healthy controls in baseline supine position. In contrast, Yataco et al. [14] had previously reported no differences in LF, HF and LF/HF in baseline supine position between CFS/ME patients and healthy controls.

During sleep, Boneva et al. [25] found shorter mean RR and reduced LF coupled with higher nor-epinephrine levels and lower aldosterone levels in plasma. The authors interpreted this as a state of sympathetic ANS predominance and neuroendocrine disturbances. Monitoring HR during nocturnal sleep in CFS/ME, Rahman et al. [26] found decreased RMSSD, HF and LF/HF ratio in CFS/ME patients compared to those healthy controls. This result is in line with Meeus et al. review [27] who concluded that HRV was only reduced during sleep in ME/CFS.

Lewis et al. [6] used frequency domain analysis to investigate the differences in autonomic dysfunction between two CFS/ME subgroups, POTS vs. non-POTS. Interestingly, they found lower LF, HF and VLF in the POTS cases. The authors did not include HRV time domain parameters, and they proposed these frequency indices as candidate biomarkers for distinguishing between these two CFS/ME phenotypes.

A relevant feature of the procedure used in the current study for recording the RR intervals is the use of 5-min records obtained on 3 different days (similar time schedule, 15–18 h) from each participant. The three values of each variable were averaged and used for the final analysis. It is likely that this method conferred robustness on the measure and, in consequence, led to a more reliable HRV value, less contaminated by everyday variables such as lifestyle habits, food, activity, sleep problem, medication, and so on.

In a recent study from our group exploring abnormalities of circadian rhythm and dysautonomia in CFS/ME, we found changes in the chronotype and symptom patterns in these patients compared with healthy controls. The findings of that study also showed a difference of almost 10 points in self-reported autonomic symptoms [28]. Using the COMPASS, which included 73 questions that assess autonomic dysfunction symptoms in CFS/ME, Newton et al. [29] concluded that an overall COMPASS cut-off score ≥ 32.5 was considered as a useful diagnostic criterion for ANS dysfunction in individuals with CFS/ME. In the current study, CFS/ME patients reported increased orthostatic intolerance, and higher scores of vasomotor, secretomotor, gastrointestinal, bladder, pupillomotor symptoms and total COMPASS scores than healthy controls, in line with previous results reported by our group [28] and others [30].

The development of telemetric devices capable of detecting and capturing R–R interval signals, together with the applications that facilitate the analysis and provide the calculation of HRV indices, may facilitate the use of these signals as biomarker in research and clinical practice. This study breaks new ground in the use of mHealth technology for the real-time analysis of cardiac variability in CFS/ME in a controlled situation. For example, mHealth has been defined as the use of mobile computing and communication technologies in healthcare and public health [31, 32]. The improvement in the speed of the processors, the smaller and longer-lasting batteries, the greater memory capacity and very precise built-in sensors enables more accurate monitoring of health parameters in real time and in natural situations [33].

Heart rate variability is considered an index of cardiac autonomic modulation. In the frequency domain, vagal (parasympathetic) activity is the major contributor to HF variability, whereas both vagal and sympathetic activity contributes to LF variability. The LF/HF ratio is considered an index of sympathovagal balance. For time domain indices, vagal (parasympathetic) activity is the main contributor to pNN50 and RMSSD, whereas SDNN is a measure of total variability, analogous to the total power index in the frequency domain [24]. Autonomic function in CFS/ME shows sympathetic hyperactivity and parasympathetic hypoactivity and this autonomic imbalance might reflect an alteration of the central control pathomechanisms.

Studying parasympathetic activity by using HF power in the frequency domain method and RMSSD in the HRV time domain method, previous studies have shown that the HF component changes after electrical vagal stimulation, muscarinic receptor blockade, and vagotomy [24]. We found decreased mean RR, SDNN, RMSSD and pNN50 in CFS/ME patients compared with healthy controls in the HRV time domain analysis of RR intervals, and the frequency domain analysis revealed decreased LF and HF, and HFnu and increased LF/HF index in CFS/ME patients. This concurrence in these HRV indices from different domains had not been previously reported.

The robust association between fatigue symptoms, anxiety-depression and HRV indices also deserves mention. All scores of fatigue symptoms (physical, cognitive, psychosocial and overall) correlated significantly and negatively with all HRV indices, except for the LF/HF ratio, which showed a positive correlation. This indicates that increased fatigue coincides clearly with a reduced variation in the time interval between consecutive heartbeats. Anxiety and depression scores were also negatively and robustly associated with time domain HRV indices and with the HF index of the frequency domain, suggesting that anxiety and depression symptoms were associated with decreased HRV. All PSQI domains except sleep latency were negatively and robustly associated with the main HRV indices, including SDRR, RMSSD, pNN50 and HF. This result is consistent with those of a previous study reporting lower nocturnal RMSSD and HF in CFS/ME patients than in healthy controls [26]. In the current study, sleep efficiency, disturbances, sleeping medication and total scores were also associated with HFnu. Interestingly, the correlations of HF and HFnu with the questionnaires scores were stronger than those of the LF and LF/HF indices. Overall, the significance of the HF and HFnu when present was greater than that of LF or LF/HF in all the correlations analysed, suggesting that HF and or HFnu may be more specific correlates of fatigue and comorbid health conditions than LF or LF/HF.

Regarding the COMPASS-31 results, four of the six domains (orthostatic intolerance, secretomotor, gastrointestinal, and pupillomotor) as well as the total score were associated with decreased HRV time domain and HF and HFnu parameters, which again corroborates the specificity of those measures as biomarker that correlate with fatigue and comorbid conditions. We stress that orthostatic intolerance, secretomotor and total scores were also associated with LF and the LF/HF ratio, but with a lower degree of significance than the other parameters mentioned above. Vasomotor and bladder domains were not associated with HRV, thus indicating certain specificity in the association between the autonomic dysfunction scored by COMPASS-31 and HRV domain parameters. With the exception of the LF/HF ratio, a significant negative association was found between all the HRV parameters and symptoms of sleep disturbances, anxiety/depression, autonomic dysfunction and, most significantly, the fatigue scores. Thus, low values for the HRV indices were associated with high scores of the clinical symptoms.

Finally, the results showed a robust relationship between the self-reported measure score of fatigue assessed by FIS-40 scale and mean RR, RMSSD and HFnu HRV indices in CFS/ME patients, but not in healthy controls. Interestingly, HF domain together with mood status (assessed by the Profile of Mood Status) and blood biomarkers (such as serum dehydroepiandrosterone sulfate levels, cortisol and TNF-α), HF improved in CFS/ME patients after a session of isometric yoga [34]. These changes may be related to a short-term fatigue-relieving effect of sitting isometric yoga and the ensuing increase in vagal nerve functioning observed due to the reduction of the heart rate and the increase in high frequency power. These results indicate the importance of the physiological parameters involved in the R–R variability, and of the assessment of fatigue severity status in individuals with CFS/ME.

## Strengths and limitations

We believe that this study is unique for several reasons: (1) the sample size is adequate in a study involving CFS/ME patients; (2) the reproducibility of HRV recordings and their technical quality were satisfactory: the use of the same location (medical lab), the same timing in the day and the week, the same device and the recordings of HRV were routinely performed by the same researcher; (3) the assessment of the ‘fatigue status’ and other core symptoms was based on patient-reported questionnaires that have been widely validated in the Spanish CFS/ME population and on an a priori threshold independent of HRV analysis; (4) from a practical point of view, we consider that the current study will help to demonstrate the relevance of HRV time- and frequency-domain analyses for the quantification and monitoring of fatigue severity and its clinical impacts in CFS/ME. However, this study has several limitations that need to be addressed: (1) the study has a cross-sectional design and the sample size is relatively small; however, there is sufficient evidence to suggest a close relationship between ANS dysfunction (assessed by HRV analysis) and most (if not all) of the multisystem symptoms in CFS/ME. The primary cause of sympathetic dysfunction in ME/CFS remains to be defined. Therefore, longitudinal large-scale studies are now warranted to elucidate the HRV changes in people with CFS/ME and also assess the natural course of cardiac autonomic dysfunction, taking advantage of the capabilities offered by mHealth technology; (2) we did not use a ‘gold standard’ for the HRV measurements (i.e. 24-h Holter ECG recorders); however, the FitLab^®^ system using the new mobile telemetric devices has proven to be an accurate and valid method for HRV measurements; (3) we did not include men with CFS/ME; this is an area we are currently exploring; (4) Short-term (5-min) HRV analysis was performed only for time- and spectral domains; perhaps other complex approaches for analysing long-term HRV over the entire 24 h (e.g., approximate and sample entropy, detrended fluctuation analysis, long-term exposure, non-linear vs. linear dynamic methods, and so on) may provide valuable additional information in relation to the prediction of fatigue status in individuals with CFS/ME.

## Conclusions

This is the first study to investigate the relationship between HRV domain indices and patient-reported measures in individuals with CFS/ME. Our findings show that HRV analysis using mHealth technology is an objective, non-invasive tool that can be useful for clinical use to predict fatigue severity, which may therefore be part of the pathogenesis in CFS/ME, since both the time- and frequency-domain indices were closely associated with ANS dysfunction in this condition. This study provides a rationale for future longitudinal studies in larger CFS/ME cohorts, including men, would be of great help to assess the impact of the HRV measurement using mHealth tech and fatigue severity (i.e. HR monitoring to prevent post-exertional malaise) both at baseline and after an exercise challenge (consecutive 2-day CPET) in patients with CFS/ME. Studies of this kind are also urgently needed to understand the pathophysiologic mechanisms due to neurohormonal imbalance, especially related with HPA axis and ANS dysfunction in the physical functioning and health-related well-being in people with CFS/ME.

## Data Availability

The dataset supporting the findings of this work are available from the corresponding author upon reasonable request.

## Abbreviations

ANS: autonomic nervous system
BMI: body mass index
CDC: Centers for Disease Control and Prevention
CFS/ME: chronic fatigue syndrome/myalgic encephalomyelitis
COMPASS-31: 31-item composite autonomic symptom score
DBP: diastolic blood pressure
FIS-40: 40-item fatigue index scale
HPA: hypothalamic–pituitary–adrenal axis
HADS: Hospital Anxiety and Depression Scale
HF: relative power of the high-frequency band (0.15–0.4 Hz)
HR: heart rate
HRV: heart rate variability
LF: relative power of the low-frequency band (0.04–0.15 Hz)
LF/HF: ratio of LF-to-HF power
MRI: magnetic resonance imaging
NCQ: Neurovegetative Complaints Questionnaire
PEM: post-exertional malaise
POTS: postural orthostatic tachycardia syndrome
POMS: Profile of Mood Status
PSQI: Pittsburgh Sleep Quality Index
pNN50: percentage of successive RR intervals that differ by more than 50 ms
RR intervals: time between two successive heart beats
SBP: systolic blood pressure
SDNN: standard deviation of NN intervals
SDRR: standard deviation of RR intervals
RMSSD: root mean square of successive RR interval differences
VLF: absolute power of the very-low-frequency band (0.0033–0.04 Hz)
